# Body Mass Index Reference Curves for Children Aged 0-18 Years in Shaanxi, China

**Published:** 2005-06

**Authors:** Lei Shang, Yong-yong Xu, Xun Jiang, Ru-lan Hou

**Affiliations:** 1 *Department of Health Statistics, The Fourth Military Medical University, Xi’an, Shanxi, Peoples Republic of China;*; 2 *Department of Pediatrics, Tangdu Hospital, The Fourth Military Medical University, Xi’an, Shanxi, Peoples Republic of China;*; 3 *Institute of Public Health, Xi’an Medical University, Xi’an, Shanxi, Peoples Republic of China*

**Keywords:** body mass index, growth reference, obesity, Chinese children

## Abstract

**Objectives:**

Health care professionals have recommended the use of age-related body mass index (BMI) to evaluate obesity in children. Until now, no age-related reference curves for BMI have been reported in China. Presented here are age-related BMI percentile curves for children aged 0~18 years in Shaanxi, China, 1995.

**Methods:**

The Third Nationwide Growth Survey was performed in 1995 and from this survey, data of the Shaanxi population were retrieved to construct the age-related BMI percentile curves. A total of 27,200 healthy children aged 0~18 years were examined for height and weight, using the standardized methods. The λ-median-coefficient of variation (LMS) method was used for curve fitting; all analyses were carried out on the basis of different sexes and areas through a special program for LMS method.

**Results:**

Median BMI increased steeply in early life, with a peak at 8 months, then declined, and then leveled off at about 6 years. The age of adiposity rebound for urban children was about two years earlier than that for rural children and one year earlier for boys than for girls. After adiposity rebound, BMI increased more rapidly in girls than in boys, and the increase in urban children was more rapid than that in rural children. As the onset of puberty, female BMI became higher than that of males, and the difference between boys and girls was larger for rural children than for urban children. The 95th, 50th and 5th percentiles for Shaanxi children were lower than those of comparable American children. Cut-off points for obesity was lower than those of international averages, suggesting the nutrition status of Shaanxi children is lower than that of children in developed countries, and has not reached the international average level.

**Conclusions:**

Using the LMS method, we constructed age-related BMI percentile curves for Shaanxi children aged 0~18 years, the first for Chinese children. Percentile curves and cut-off points for obesity can be used as a reference for assessing the nutrition status of Shaanxi children aged 0~18 years. The identified gender and residency differences may serve as guides to an understanding of the cause and prevention of obesity.

## INTRODUCTION

Obesity is a serious public health problem among children and adolescents. It is believed to be one of the most serious nutritional problems in the world, and has been identified as a causal factor for a variety of medical problems, including insulin resistance, abnormal lipids and lipoproteins, elevated blood pressure, and adults’ morbidity and mortality. Thus, obesity in childhood will lead to a lower quality of life and higher medical costs in the future. In recent years, early childhood obesity has risen rapidly in developing countries. In the United States, during the past 30 years, the prevalence of overweight [defined as body mass index (BMI, in kg/m^2^) ≥85th percentile of NHANES Ш] in children aged 6~11 years has increased from 15.2% to 22.3% (1). Among North American indigenous peoples the prevalence of obesity of similar ages (using BMI 85th percentile of NHANES П as a cutoff) was 30~40% in boys and 35~43% in girls (2). In China, the occurrence of obesity (defined as weight-to-height standard of NCHS/CDC, weight excess weight-for-height≥10~19% is overweight, and weight excess weight-for-height≥20% is obesity) for children aged 0~7 years is 2.2% for boys and 1.9% for girls and the occurrence of obesity for children aged 0~7 years is 4.2%. For children aged 7~12 years, the occurrence of obesity is about 1.5~5.5%, which is higher in urban areas and increasing rapidly. The increase in the past ten years is about 93.4%, and the average annual increase is 9.3% (3).

Shaanxi Province is located in the northwest of China, with a population of 35 million, of whom 35% are children and adolescents, and covers an area of 0.2 million km^2^. In recent years, Shaanxi has emerged as a prosperous economic and cultural center, with a GNP of US$ 456 per capita, and a rural gross income of US$ 167.4 per capita. The ten-year rate of increase in GNP per capita was 281%, that of farm gross income was 250%, and all economic indices of Shaanxi are similar to those of the other parts of the country (4). Compared with others, children growth and development status in Shaanxi is at an average level (5), which can be seen from Table 1. The development of Xi’an children ranks 15th for boys and 16th for girls among the 28 provincial capital cities of China, which the very sample region for the Second and Third National Growth Surveys. Therefore, the physical development status of Shaanxi children and adolescents may be representative of the country as a whole.

**Table 1:** Rank of 18-years-old children’s development level among 28 cities in China

City	Height		Weight		Breast circumference		Development score		Rank	
	boys	girls	boys	girls	boys	girls	boys	girls	boys	girls

Beijing	171.04	159.15	59.88	51.48	87.18	80.07	105.17	103.04	1	1
Jinan	170.17	158.21	59.35	51.46	86.44	79.65	103.82	102.16	2	4
Harbin	170.07	158.25	58.45	50.04	86.35	79.12	103.15	100.86	3	9
Shijiazhuang	169.93	157.71	59.29	50.71	85.09	77.89	102.65	100.26	4	15
Shenyang	170.44	159.14	57.46	50.75	85.19	79.66	101.96	102.27	5	2
Hefei	169.79	157.77	56.92	49.81	86.16	80.36	101.93	101.09	6	8
Tianjing	169.96	158.56	57.73	50.96	85.22	79.38	101.87	101.86	7	5
Nanjing	169.39	158.27	57.30	50.68	86.04	80.71	101.84	102.82	8	3
Nanzhou	169.39	157.53	56.29	49.95	85.79	79.17	101.06	100.34	9	13
Shanghai	170.76	158.40	56.94	49.98	83.36	78.47	100.93	100.52	10	12
Huhehaote	167.95	157.56	56.94	51.36	84.53	79.58	100.33	101.61	11	6
Hangzhou	169.97	158.51	57.05	50.19	83.71	79.22	100.25	101.19	12	7
Urmqi	169.51	158.58	56.92	50.45	84.89	78.27	100.20	100.84	13	10
Changchun	169.95	158.58	56.73	50.03	83.67	77.69	100.19	100.28	14	14
Xian	168.57	157.17	56.02	50.20	84.87	79.09	99.77	100.24	15	16
Yinchuan	169.04	158.25	56.23	50.92	83.71	77.51	99.37	100.54	16	11
Zhengzhou	169.00	158.01	56.94	50.27	82.91	78.02	99.22	100.21	17	17
Kunming	167.54	156.43	54.92	48.94	84.88	79.19	98.50	98.89	18	22
Taiyuan	169.10	156.74	56.08	49.63	82.18	78.84	98.27	99.39	19	21
Changsha	166.50	156.45	55.25	49.85	84.53	79.50	97.83	99.92	20	19
Nanchang	166.78	156.13	55.77	49.23	83.68	78.14	97.73	98.65	21	23
Wuhan	167.50	156.81	55.26	50.11	82.80	78.72	97.48	99.72	22	20
Fuzhou	168.62	157.65	54.04	47.18	83.20	74.79	97.45	95.83	23	27
Xining	167.42	157.41	54.79	50.89	82.13	77.48	96.45	99.95	24	18
Guangzhou	166.66	156.73	53.73	47.95	83.54	79.51	96.33	98.56	25	24
Chengdu	165.88	154.80	54.01	48.03	82.39	78.26	95.44	96.62	26	25
Nanning	165.28	154.43	53.71	47.45	82.44	77.96	95.03	95.78	27	28
Guiyang	165.16	153.93	53.39	48.52	81.95	77.92	94.13	96.20	28	26

## SUBJECTS AND METHODS

### Subjects

The reference sample of children was obtained by combining data of 2 individual surveys. The data for children under 7 years in Xi’an came from the Third National Cross-sectional Growth and Development Survey, including 8800 (50%) boys and 8800 (50%) girls; and 8800 (50%) from the urban and 8800 (50%) from the rural areas. These children were classified into 22 age-groups. Except for the first group of 0~3 days and the last group of 6.0~6.99 years, the rest were divided into monthly (1.0~5.9months), bimonthly (6.0~11.9 months), 3-monthly (12.0~23.9 months) and 6-monthly (2.0~5.99 years) intervals. The sample size was 200 for each sex/age group in both the urban and the rural groups. Children with deformities or diseases severely affecting their growth and development and general health were not included in the study. In the group aged 0~3days, babies with birth-weight under 2500 g, premature babies and twins were excluded. The Xi’an Maternal and Child Health Station was appointed to organize the survey, under the direction of the Beijing Steering Committee, the nine coordinating study sub-groups on the Third National Growth and Development Survey of Children (GDSC).

The data for children of Shaanxi aged 7~18 years were from the Third National Cross-sectional Study on Chinese Students’ Constitution and Health. All 9600 subjects were elementary and middle school students, including 4800 boys and 4800 girls, and 4800 from the urban and 4800 from the rural areas. The samples were divided into 12 age groups at yearly intervals from 7 to 18 years old. There were 200 subjects in each age-sex group. All subjects were healthy students and passed a general physical examination aimed at excluding diseased subjects. The Shaanxi Research Group on Students’ Constitution and Health was appointed to organize the survey, under the direction of the National Research Group on Students’ Constitution and Health.

For each subject, his/her age was calculated from his/her birthday (e.g. 7-years-old including 7~7.99 years). The height and weight were measured by specially trained technicians or nurses, and the techniques of measurement were the same as those in 1975 (6, 8). The measurement of subjects was conducted between 8.00 am and 4.00 pm every day in a 3-month period between April and June, 1995.

### Statistical methods

Raw data was processed by SPSS 11.0. (7) Summary percentile curves were fitted to the data using the LMS method and penalized likelihood (9, 10, 11, 13), which involved normalizing the data at each age using a Box-Cox power transformation. The percentiles at each age can thus be summarized in terms of the Box-Cox power needed to make the distribution normal (called L), and the median (M) and coefficient of variation (S) of the distribution. The fitting process ensured that values of L, M and S changed smoothly with age so that they serve as the smooth curves plotted against age. The three quantities provide the required percentiles and expressed by the following formula.

C100α (t)=M (t) [1+L (t) S (t)Zα] 1/L (t)

In this formula C100α (t) is the percentile curve plotted against age t, Zα is the normal equivalent deviate for the percentile (for example when α=0.97, corresponding to the 97th percentile, Zα=1.88), and L (t), M (t), and S (t) are the fitted smooth curves plotted against age. Since these curves in our experiment were smooth, the resulting percentile curve was smooth as well.

There are three main advantages of this approach. Firstly, it estimates extreme percentiles more efficiently than the simpler “sort and count” procedure, and it allows skewness in the distribution. Secondly it can generate any required percentiles in addition to the conventional set of seven. Thirdly, percentiles constructed by LMS method allow data to be converted directly to Zα, represented by the formula:

SD score=measurement/MtLt−1S t L t

The quantities L, M and S are natural cubic splines with knots at each ti, and are estimated by maximum penalized likelihood. The complexity of each spline is measured by its equivalent degrees of freedom (e.d.f). The e.d.f. for each Curve is analogous to the degrees of freedom of a polynomial, and change from 2 upwards. The lower bound of 2 corresponds to an infinitely smoothed curve, that is a straight line, while larger e.d.f. values correspond to progressively rougher spline curves. In this study, the chosen LMS e.d.f. for urban boys is 4, 12 and 7 respectively, for urban girls 3, 10 and 7, for rural boys 4, 12 and 7, and for rural girls 4, 12 and 7. This process does not work if the M curve is non-monotonic or the median curve of BMI is non-monotonic, so age is transformed to loge (age+0.27) before fitting.

We used the LMS program provided by T. J. Cole and Huiqi Pan to carry out our analyses (Cole *et al*, 1992). Excel 2000 was used to draw the figures (12).

## RESULTS

**Figure 1:**
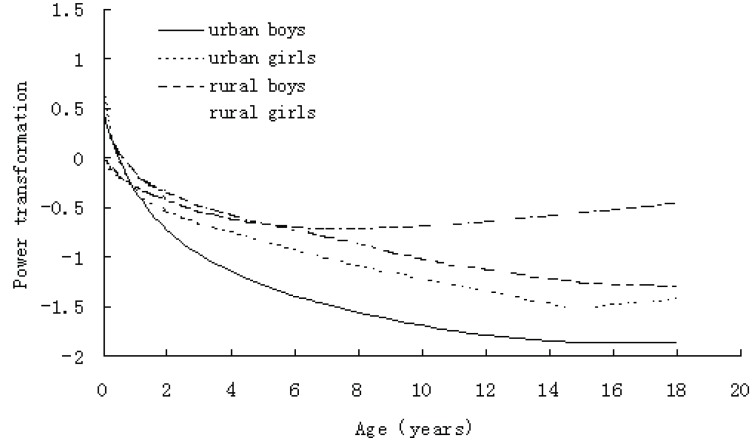
Degree of skewness (L curve) of BM in Shaanxi children.

The L curves (Figure 1) measure the skewness of the BMI distribution, a value of 1 indicates normality and values smaller than 1 representing progressively greater skewness. At birth BMI was already somewhat skewed, but during the first year when BMI rose steeply, the degree of skewness also increased sharply. Subsequently, it changed little, although the skewness of urban girls became decreased from 16 years of age. It can be seen from Figure 1 that the skewness for rural children was bigger than for urban children, that the skewness for girls was bigger than for boys after 2 years of age, and that the skewness for rural girls was below that of rural boys after 6 years of age. Differences between areas and sexes became greater as adolescence started. This degree of skewness was reflected in the spacing of the BMI percentiles, with the top percentile channel in the four figures wider than the bottom percentile channel at all age.

**Figure 2:**
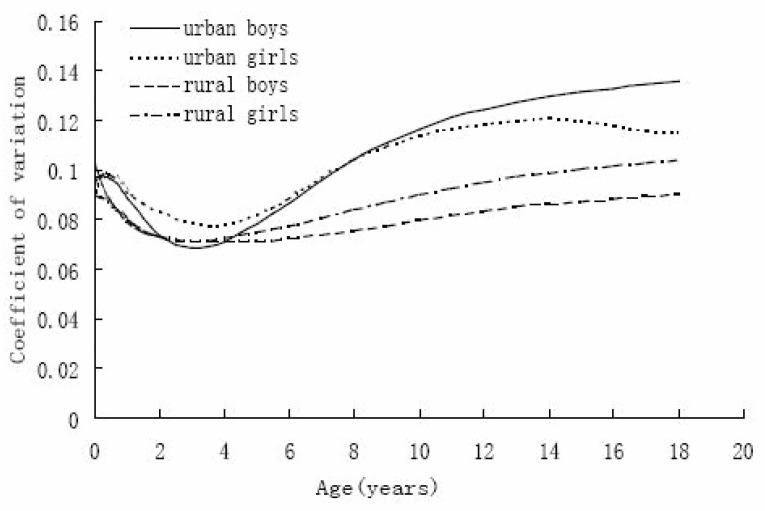
Coefficient of variation (S curve) of BM in Shaanxi children.

The S curve (Figure 2) defines the co-efficient of variation of BMI. The variability was about 0.10 in infancy, falling to below 0.08 and then rising to a peak of 0.14~0.12 for urban children and rising a little for rural children in adolescence. From Figure 2 we noted that the variability among urban children was greater than that of rural children, and from 4 years on, the variability among rural girls was greater than that of rural boys. But the S curve for urban boys intersected that of urban girls at about 8 years; the variability for urban boys increased compared to that of urban girls upon adolescence, and after 16 years of age, variability among urban girls fell slightly.

**Figure 3:**
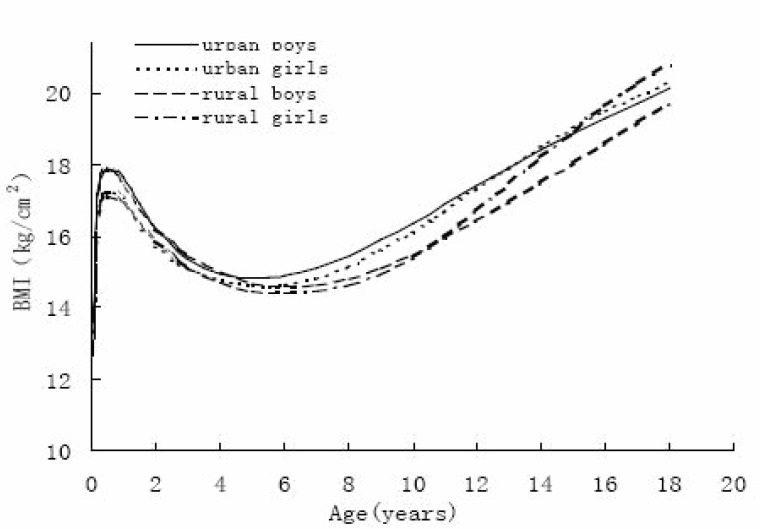
Median (M curve) of BM in Shaanxi children.

The M curve is the median of BMI. The changes in median BMI by age was on the whole very similar in the two sexes and areas. BMI increased steeply in one’s early life, to a peak of about 17.00 kg/m^2^ at 8 months, then it declined, and then leveled off at 6 years when BMI is about 14.60 kg/m^2^. This dip in BMI is called adiposity rebound (14), The age at adiposity rebound (Figure 3) for urban children was about two years earlier than for rural children and one year earlier for boys than girls. In infancy, the median BMI appears at the same age for the four groups. After the adiposity rebound, BMI increased more rapidly in girls than in boys, and increased more rapidly among urban children than their rural counterparts. Median curves for boys and girls intersected at age thirteen for urban subjects and at age eleven for rural ones. After the curves intersected, female BMI was higher than male, and the difference between boys and girls was larger in rural areas than in urban areas. At the end of puberty, rural female BMI was highest and rural male BMI was lowest among the four groups.

**Figure 4:**
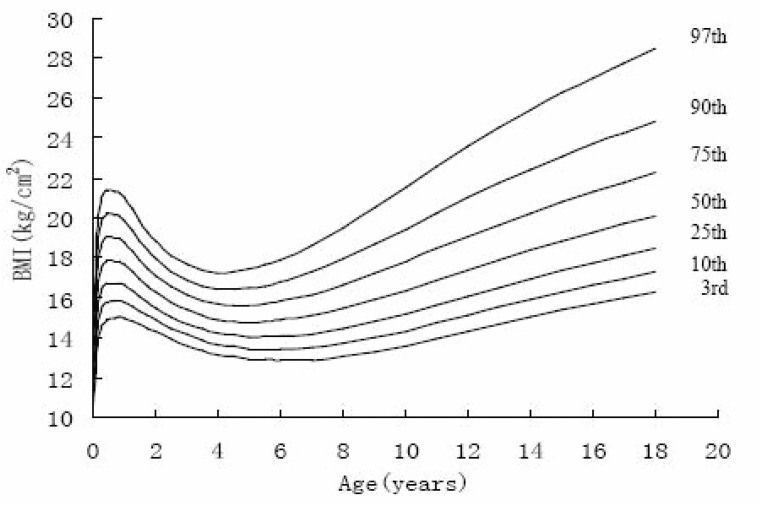
The 3rd, 10th, 25th, 50th, 75th, 90th, 97th percentile curves of BM of urban boys in Shaanxi from 0-18 years.

Figure 4~Figure 7 gives BMI reference percentile curves for each group in the seven percentile formats. Percentiles are the conventional 3rd, 10th, 25th, 50th, 75th, 90th and 97th. As figures showed, the shape of the percentile curves were almost the same among the four groups, although at the end of puberty, urban girls upper percentile curves (97th, 90th) declined after 16 years old, which indicated there existed a subjective loss of weight among urban girls. The age at adiposity rebound was later on the lower than the higher curves. In infancy, all the percentiles’ peak appeared at the same age. After adiposity rebound, upper percentiles increased more rapidly and earlier than lower percentiles. Regarding the difference between percentiles, upper percentile differences were greater than the bottom percentile differences for all ages, and urban group percentile differences were greater than those of rural groups, indicating the skewness for urban groups was greater than for the rural groups.

**Figure 5:**
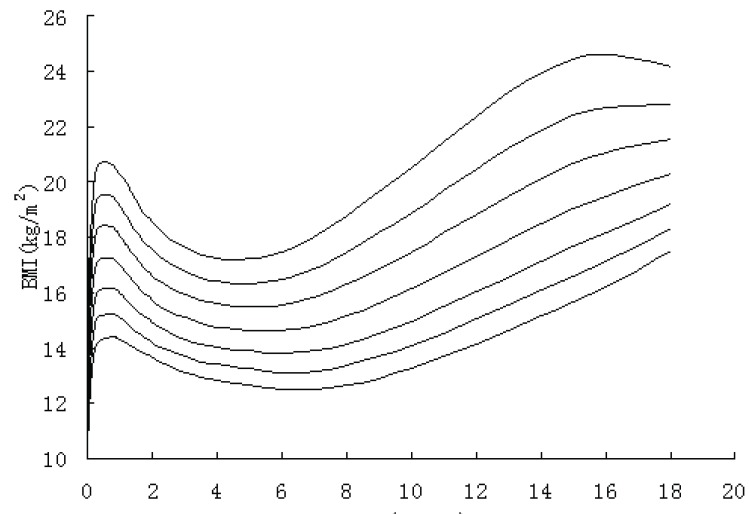
The 3rd, 10th, 25th, 50th, 75th, 90th, 97th percentile curves of BM of urban girls in Shaanxi from 0-18 years.

**Figure 6:**
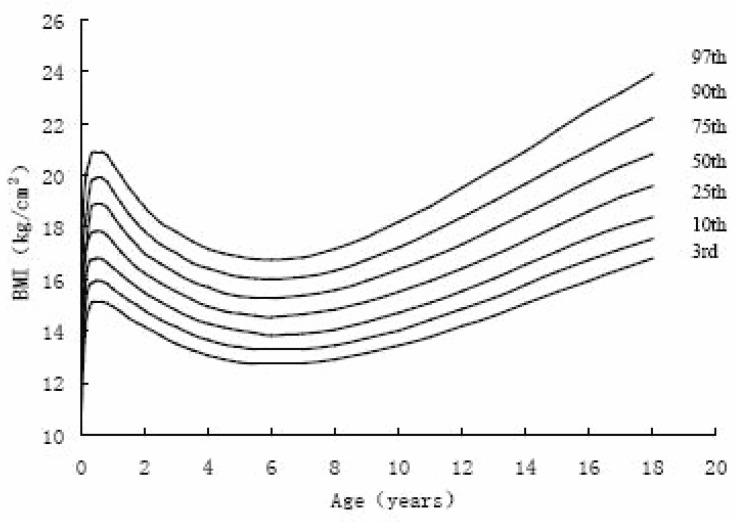
The 3rd, 10th, 25th, 75th, 90th, 97th percentile curves of BM of rural boys in Shaanxi from 0-18 years.

**Figure 7:**
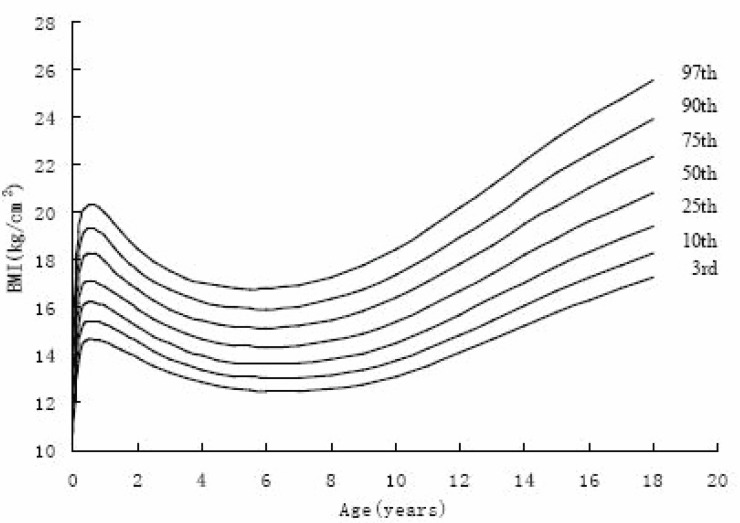
The 3rd, 10th, 25th, 50th, 75th, 90th, 97th percentile curves of BM of rural girls in Shaanxi from 0-18 years.

Table 2 shows the proportions of the data between the seven percentiles, and there was an excess of less than 1 per 1000 (3.1 per cent versus 3 per cent) beyond the extreme percentiles, while the tails beyond Zα=±2 exactly matched the expected sizes of 2.4 percent. It is clear that apart from random errors the observed percentiles were close to the expected values, and the distribution was reasonably normal.

**Table 2:** Percentage of the measurements, expressed as SDS, falling in the 8 channels defined by the 7 percentiles. The expected percentages based on a normal distribution are also shown

	1	2	3	4	5	6	7	8

Urban boys	2.9	7.1	14.9	25.2	25.2	14.9	6.8	3.0
Urban girls	3.1	6.8	14.8	25.0	25.1	15.2	6.9	3.1
Rural boys	3.2	6.9	15.1	24.9	24.9	15.3	6.8	2.9
Rural girls	2.9	7.2	14.9	24.9	25.2	14.9	7.1	2.9
Expected	3	7	15	25	25	15	7	3

To test the goodness of fit of the model, data were grouped at yearly intervals, and the proportion falling below the 3rd, 50th and 97th percentiles (that is Zα below -1.88, 0 and 1.88 respectively) was calculated. The observed numbers falling below the percentiles were compared with the expected numbers to produce a χ^2^ statistic with 1 degree of freedom for each age and percentile group, and the distribution of those individual χ^2^ statistics was examined. For urban boys the χ^2^ values varied between 0 and 2.96 with a median of 0.56, and the spread of values was compatible with a standard χ^2^ distribution with 1 degree of freedom. The same was true for the rural boys, and both the urban and rural girls. The value varied between 0 and 4.17 with a median of 0.36 for urban girls, 0 and 4.65 with a median of 0.42 for the rural boys, and 0 and 3.71 with a median of 0.18 for the rural girls. The spread of the values showed no distinct patterns with age for either sex or areas, as showed by significance test. There was no difference between fitted and actual distributions in each group.

## DISCUSSIONS

The BMI percentile curves presented in this paper are the first to be developed for children aged 0~18 years in China. They were based on recently collected and representative data which were taken from a national survey conducted in 1995. They may or may not reflect the current situation in China. The percentiles are similar in general shape to those published for French, American and British children, but the percentiles for Shaanxi children aged 0~18 years are lower than that for American children (Refer to Table 3). This indicates the difference in percentiles between Chinese children and children in the developed countries. The France charts were based primarily on data from 1970s (15). The American charts were based primarily on data from NHANESl (16; The First National Health and Nutrition Examination Survey, 1971 to 1974). These data showed the nutrition statues and figure of America and France in the 1970s. Since then both childhood and adult obesity has increased greatly (13, 17). This may indicate the nutrition status is significantly different between Chinese children and children in developed countries.

**Table 3:** Comparison of Shaanxi children’s 95th 50th and 5th BMI percentiles with American’s

Age (y)	America						Shaanxi											
	boys			girls			urban boys			urban girls			rural boys			rural girls		
	95th	50th	5th	95th	50th	5th	95th	50th	5th	95th	50th	5th	95th	50th	5th	95th	50th	5th

1	19.9	17.2	14.6	19.3	16.6	14.7	19.7	16.8	14.1	19.6	16.4	13.8	19.8	17.0	14.4	19.2	16.4	13.9
2	19.0	16.5	14.4	18.7	16.0	14.3	19.0	16.4	14.0	18.9	16.0	13.7	18.8	16.3	14.0	18.4	15.9	13.7
3	18.4	16.0	14.0	18.3	15.6	13.9	18.4	15.9	13.8	18.2	15.5	13.4	17.9	15.6	13.5	17.6	15.3	13.2
4	18.1	15.8	13.8	18.2	15.4	13.6	17.9	15.4	13.4	17.5	15.0	13.0	17.4	15.2	13.3	17.1	14.8	12.8
5	18.0	15.5	13.7	18.3	15.3	13.5	17.5	15.0	13.0	17.1	14.7	12.7	16.9	14.8	12.9	16.9	14.6	12.6
6	18.1	15.4	13.6	18.8	15.3	13.3	17.2	14.7	12.6	17.1	14.6	12.6	16.7	14.6	12.7	16.7	14.4	12.4
7	18.9	15.5	13.6	19.7	15.5	13.4	17.7	14.7	12.5	17.6	14.8	12.7	16.8	14.7	12.8	16.7	14.4	12.4
8	19.7	15.7	13.7	21.0	16.0	13.6	18.5	15.0	12.6	18.2	15.1	12.8	17.1	14.9	13.0	17.0	14.5	12.5
9	20.9	16.0	14.0	22.7	16.6	14.0	19.6	15.5	12.8	19.4	15.6	13.0	17.6	15.2	13.2	17.5	14.8	12.6
10	22.2	16.6	14.2	24.2	17.1	14.3	20.7	16.1	13.2	21.0	16.2	13.3	18.3	15.5	13.5	18.3	15.2	12.8
11	23.5	17.2	14.6	25.7	17.8	14.6	22.2	16.6	13.5	22.5	16.8	13.6	18.9	15.8	13.7	19.1	15.7	13.1
12	24.8	17.8	15.1	26.8	18.3	15.0	23.3	17.0	13.9	23.9	17.5	14.1	19.5	16.1	14.0	20.3	16.5	13.6
13	25.8	18.4	15.6	27.9	18.9	15.4	24.1	17.4	14.2	25.1	18.3	14.8	19.9	16.5	14.3	21.7	17.5	14.3
14	26.8	19.1	16.1	28.6	19.4	15.7	24.6	18.0	14.7	25.5	18.9	15.3	20.6	17.1	14.8	22.9	18.5	15.1
15	27.7	19.7	16.6	29.4	19.9	16.1	25.2	18.6	15.2	25.4	19.4	15.8	21.3	17.9	15.5	23.6	19.3	15.8
16	28.4	20.5	17.2	30.0	20.2	16.4	25.9	19.2	15.6	25.2	19.8	16.3	22.3	18.7	16.1	24.0	19.9	16.4
17	29.0	21.2	17.7	30.5	20.7	16.9	26.4	19.7	15.9	25.1	20.1	16.6	23.0	19.2	16.4	24.4	20.5	17.0
18	29.7	21.9	18.3	31.0	21.1	17.2	26.8	20.2	16.2	24.7	20.2	16.7	23.8	19.8	16.7	24.5	21.0	17.5

Table 4 gives the percentile and Zα for obesity by sex between 0~18 years, defined to pass through BMI of 25 and 30 kg/m^2^ at age 18 (19). BMI of 25 and 30 kg/m^2^ are overweight and obesity cut off points for adults (18). As for overweight percentiles, Shaanxi was the lowest among 7 countries. As for obesity percentiles, Shaanxi was among the three lowest, reflected that children’s overweight and obesity were related with economic status of it’s countries obviously, percentile for overweight and obesity in developed countries were higher than that in developing countries.

**Table 4:** Comparing the percentiles and Z scores for overweight and obesity by sex between 0~18 years, defined to pass through BMI of 25 and 30 kg/m^2^ at age 18 among different countries

Country	overweight						obesity					
	boys			girls			boys			girls		
	percentile Z	score %	above cut-off point	percentile Z	score %	above cut-off point	percentile Z	score %	above cut-off point	percentile Z	score %	above cut-off point

Brazil	95.3	1.68	4.7	84.8	1.03	15.2	99.9	3.10	0.1	98.0	2.10	2.0
Britain	90.4	1.30	9.6	88.3	1.19	11.7	99.1	2.37	0.9	98.8	2.25	1.2
Hong Kong	88.3	1.19	11.7	90.2	1.29	9.8	96.9	1.86	3.1	98.2	2.10	1.8
Netherlands	94.5	1.60	5.5	93.5	1.52	6.5	99.7	2.71	0.3	99.7	2.73	0.3
Singapore	89.5	1.25	10.5	93.0	1.48	7.0	98.3	2.12	1.7	99.0	2.33	1.0
American	81.9	0.91	18.1	83.5	0.97	16.5	96.7	1.84	3.3	96.0	1.76	4.0
Shaanxi	95.3	1.67	4.7	94.5	1.60	5.5	99.5	2.60	0.5	99.8	2.85	0.2

From Table 5, it can be seen that cut-off points for overweight and obesity for Shaanxi children aged 0~18 years were lower than those for international children (19), the cut-off point difference for obesity between Shaanxi and international children and adolescents was greater than that for overweight. These figures indicate Shaanxi children tend to be thinner than the international average, but the figure distribution for the whole population is even. Shaanxi children and adolescents’ nutrition status has not reached the international average level.

**Table 5:** Cut-off points for BMI for overweight and obesity by sex between 0~18 years, defined to pass through BMI of 25 and 30 kg/m^2^ at age 18

Age (years)	overweight				obesity			
	boys		girls		boys		girls	
	Shaanxi	International	Shaanxi	International	Shaanxi	International	Shaanxi	International

0	14.93		14.67		16.17		16.33	
1	20.20		19.50		21.95		21.81	
2	18.39	18.41	17.95	18.02	19.84	20.09	19.94	19.81
3	17.44	17.89	17.11	17.56	18.79	19.57	18.96	19.36
4	16.98	17.55	16.71	17.28	18.35	19.29	18.55	19.15
5	16.88	17.42	16.60	17.15	18.37	19.30	18.55	19.17
6	17.02	17.55	16.71	17.34	18.67	19.78	18.83	19.65
7	17.36	17.92	17.00	17.75	19.22	20.63	19.37	20.51
8	17.87	18.44	17.47	18.35	20.00	21.60	20.12	21.57
9	18.50	19.10	18.07	19.07	20.91	22.77	21.00	22.81
10	19.19	19.84	18.78	19.86	21.89	24.00	22.01	24.11
11	19.96	20.55	19.60	20.74	22.99	25.10	23.12	25.42
12	20.70	21.22	20.46	21.68	24.03	26.02	24.25	26.67
13	21.45	21.91	21.34	22.58	25.08	26.84	25.39	27.76
14	22.20	22.62	22.22	23.34	26.12	27.63	26.51	28.57
15	22.93	23.29	22.99	23.94	27.14	28.30	27.48	29.11
16	23.64	23.90	23.74	24.37	28.13	28.88	28.41	29.43
17	24.32	24.48	24.40	24.70	29.08	29.41	29.25	29.69
18	25.00	25.00	25.00	25.00	30.00	30.00	30.00	30.00

Since 1975, three large surveys on children’s and adolescents’ constitution and health, growth and development have been carried out in China. Growth and development references for Chinese children and adolescents based on these surveys have been established, but these references are presented as or percentile grades, and age-related percentile curves from birth to adult have not been seen. Height for age, weight for age, weight for height, head-circumference, chest-circumference and sitting-height are mostly involved in these growth standards, but BMI reference has not been reported in China. We usually use weight ratio and skinfold to assess obesity and thinness in clinical and child health care practice, and BMI is seldom used in identifying obesity in children and adolescents. Because China. is a developing country, a distinctive difference in children’s nutrition and physiques exists between China and developed countries in the world. Therefore, it is improper to use other countries’ BMI references to assess Chinese children’s constitution and nutrition states. Percentile curves presented in this paper based on the data from Shaanxi can not only be used to assess nutrition statues for children aged 0~18 years in Shaanxi, it can also be used as a reference for other areas and the whole country.

## CONCLUSION

In conclusion, this paper makes the first attempt to construct BMI percentile curves for children over the age range of birth to 18 years in Shaanxi, China, on the basis of the data of the Third Nationwide Growth Survey carried out in 1995. The percentile curves were derived by using Cole’s LMS method, which adjusts the BMI distribution for skewness and allows BMI in individual subjects to be expressed as an exact percentile or SD score. The table and curves presented in this paper will allow pediatricians to assess the nutrition status of a child and determine the relative rank of BMI for patients in clinical setting and epidemiological studies.
